# Social support and the incidence and persistence of depression between antenatal and postnatal examinations in Turkey: a cohort study

**DOI:** 10.1136/bmjopen-2014-006456

**Published:** 2015-04-01

**Authors:** Vesile Senturk Cankorur, Melanie Abas, Oguz Berksun, Robert Stewart

**Affiliations:** 1 Department of Psychiatry, Ankara University Faculty of Medicine, Ankara, Turkey; 2 King's College London (Institute of Psychiatry), London, UK

**Keywords:** EPIDEMIOLOGY

## Abstract

**Objectives:**

This study aims to measure incidence and persistence of depression and to investigate the influence of self-reported antenatal social support and traditional/nuclear family structure on incidence and persistence of depression between the third trimester of pregnancy and following childbirth. We hypothesised that lower antenatal social support would be associated with incidence and persistence of case-level depressive symptoms and the family structure would have an effect on the incidence and persistence of depressive symptoms.

**Settings:**

The cohort study described here was carried out in and around Ankara the capital of Turkey, because of the considerable heterogeneity of the population in terms of traditional Middle Eastern and ‘modern’ Western lifestyle and social environment. Samples were drawn from 20 urban and rural antenatal clinics (mainly primary care settings) within the geographic catchment.

**Participants:**

Of 730 women recruited in their third trimester, 578 (79.2%) were re-examined between 2 and 6 months after childbirth. Exclusion criteria were as follows: aged younger than 18 years, illiteracy, significant health problems and refusal to participate.

**Primary and secondary outcome measures:**

Close Persons Questionnaire items enquired about relationships with the husband, mother and mother-in-law and depression was ascertained using the Edinburgh Postnatal Depression Scale at the each assessments.

**Results:**

In those followed, onset of postnatal depression occurred in 13.9% and persistence of antenatal depression in 49.7%. After adjustment, worse emotional support from the mother-in-law was significantly associated with postnatal depression incidence (OR=0.93, 95% CI 0.87 to 0.99) and worse emotional support from the husband with postnatal persistence (OR=0.89, 95% CI 0.83 to 0.96) of antenatal depression. Family structure was not a risk or modifying factor.

**Conclusions:**

The incidence and persistence of postnatal depression in this Middle Eastern cohort were comparable to international findings. Certain family relationships predicted incidence and persistence of postnatal depression but no role of traditional/nuclear family structure was found.

**Strengths and limitations of this study**

- To our knowledge, this is the first prospective study of perinatal depression and family support in a Middle Eastern society.
- This study shows that the relationships between women and their mother in-law remain important in Turkish culture, whether the woman is living in a nuclear or extended family setting.
- Considering the outcome, the Edinburgh Postnatal Depression Scale has been widely used in international research; however, it should be borne in mind that it is a screening instrument, measuring number of depressive symptoms and not seeking to define specific depression syndromes or to apply diagnostic criteria.

## Background

Perinatal depression is a major health issue for many women from diverse cultures, although is most often investigated in the postnatal rather than antenatal period.^1^ However, postnatal depression is often a continuation of antenatal depression, with high persistence (37–46%) and relatively low incidence (5–7%) between antenatal and postnatal periods.^2^ ^3^ Findings suggest that the continuation of depression from the antenatal to postnatal period is an important health issue but research has been mainly carried out in Western settings,^3^ and some studies from developing countries have reported high perinatal depression persistence.^4^ In a prospective cohort study conducted in Iran, where 1291 women in their third trimester were followed up to 6–8 weeks postpartum,^4^ incidence and persistence of depression were 20.1% and 49.6%, respectively.^4^ Both incidence and persistence of perinatal depression may have important later implications. Deave *et al*^5^ in a large prospective community-based study, followed women throughout pregnancy and into the child's adolescent years, finding that persistent depression during pregnancy was associated with a 50% increase in the odds of developmental delay at 18-month assessment, after adjustment. However, Brennan *et al*^6^ found no association between severity or chronicity of depressive symptomatology and child behaviour.

Social support is a commonly identified risk factor in higher and lower income settings, although its impact is likely to vary between cultures and different family/social contexts. Family context differs substantially between cultures; in particular, nuclear family settings are common in Western countries, while extended family structures are more prevalent elsewhere. In extended family settings where women live with their husband and parents-in-law, there are a wider range of relationships with potential influences on perinatal depression. In a recent review of risk factors for perinatal disorder in low-income and middle-income nations, social and economic disadvantage, crowded households and rural residence, were highlighted as most salient, as well as problems associated with the intimate partner relationship such as rejected paternity, lack of support, or excessive alcohol use and other relationship issues including lack of help from the mother-in-law, insufficient social support more generally, or maternal rural residence.^7^ Living in a nuclear rather than traditional household was also concluded as a risk factor.^7^ A recent study also concluded that receiving social support was associated with a reduced the risk of postnatal depression,^8^ and another study found that non-partner social support was particularly and independently associated with postnatal depressive symproms.^9^ However, few studies to date in low-income and lower middle-income countries have assessed social support using a formal research instrument, and none to our knowledge have investigated social support as a predictor of the incidence or persistence of perinatal depression, or have investigated the effect of traditional or nuclear family structure on these associations.^10^ Given the potential role of social support in perinatal depression, the potential influence of family structure on social support, and the lack of evidence on family structures outside Western settings, there is a clear need for further research in this area, as these issues are likely to be important determinants of targets for intervention strategies.

Evaluation of different family structures is important in women's mental health research because of the rapid ‘Westernisation’ of families occurring in many settings around the world. Turkey is almost unique as a nation in the length of time over which modern Western (‘nuclear’) and traditional Middle Eastern (‘extended’) family structures have coexisted. In the context of an ongoing prospective study of perinatal depression and child development carried out in Ankara, we analysed data from examinations carried out during the third trimester of pregnancy and 2–6 months postpartum to investigate incidence and persistence of perinatal depressive symptoms between those two periods and the influence of family relationships and structure on these transitions. We focused on three key relationships of high importance for women in Middle Eastern settings: that with the husband, the mother and the mother-in-law. Having previously found that lower reported quality of these relationships (particularly the spouse and mother-in-law) were associated contemporaneously with depressive symptoms in the antenatal period,^11^ we hypothesised that they would also be associated with incidence and persistence of case-level depressive symptoms between the antenatal and postnatal period. We had also found at baseline that associations between lower quality spouse relationship and antenatal depressive symptoms were stronger in traditional compared to nuclear family settings,^11^ and hypothesised that this would also be observed prospectively for incidence and persistence of depressive symptoms.

## Methods

### Setting

The cohort study described here was carried out in and around Ankara, the capital of Turkey, because of the considerable heterogeneity of the population in terms of traditional Middle Eastern and ‘modern’ Western lifestyle and social environment. ‘Ankara’ here includes both central urban and semirural locations. In common with other Turkish cities, it has experienced rapid expansion and immigration; for example, in 2000, 47% of residents in Ankara were found to have been born elsewhere.^12^ Many young women living in urban districts have migrated as students or working adults and live a long distance away from their parents. On the other hand, in the surrounding more rural districts, women will be more likely to be living close to their family with traditional ties and expectations.

### Participants

In total there are 18 Mother and Child centres in the urban area of Ankara and 14 in surrounding but accessible semirural regions. The initial plan was to sample equal numbers of urban and semirural centres. Although at the start of the study 10 urban and 10 rural centres were selected at random to comprise the sampling frame, there were many attendees in some centres but very few in others. Furthermore, during recruitment it was realised that these centres did not capture the whole Ankara population. Therefore, it was decided also to include one university hospital (Ankara University School of Medicine Gynaecology and Obstetrics Department) and one gynaecology and obstetrics hospital (The Ministry of Health Zekai Tahir Gynaecology and Obstetrics Hospital) as recruitment centres and, in order to improve recruitment and increase statistical power, accepting the fact that the centres would be a convenience rather than random sample. There are a total of five university hospitals which run gynaecology and obstetrics services. There are also three state gynaecology and obstetrics hospitals which mainly serve the general population in antenatal and postnatal periods. These hospitals are also usually used for hospital deliveries. Recruitment and baseline examinations have been also previously described.^9^ In summary, samples were drawn from 20 urban and rural antenatal clinics within the geographic catchment. These clinics were purposively selected to maximise population heterogeneity as it was not feasible to carry out a formal random sampling process. In participating clinics, attempts were made to interview all women attending routine third trimester antenatal examinations within the study period from December 2007 to August 2008. Usual clinic attendance is at around 32 weeks, and attendances for routine perinatal services are very high in Ankara: for example, attendance levels for measles and BCG vaccinations in the same clinics are 93% and 92% respectively.^13^ In Turkey as a whole, the percentage of pregnant women obtaining antenatal care was estimated to be 63% in 1993, 80% in urban areas, and 96% in women with secondary school education.^14^ After approach, agreement and written informed consent, a brief interview was administered by trained graduate-level research workers at the time of clinic attendance. Women with depressive symptoms were not formally re-evaluated clinically. However, women with moderate or severe depressive symptoms and wishing treatment were referred to their general practitioner or to a psychiatrist. Exclusion criteria were as follows: aged younger than 18 years, illiteracy, significant health problems and refusal to participate. Although participants were enrolled to this study through the outpatient clinics where healthy pregnant women had routine checks, women were asked if there were any significant health problems such as diabetes mellitus, hypertension and thyroid dysfunction. Participants were followed up after childbirth and interviews were held in participants’ homes between 2 and 6 months after childbirth, this range of time having arisen because of difficulties in tracing and re-interviewing some of the sample due to a local re-allocation of housing (which also gave rise to attrition as described in the Results).

### Measurements

Depressive symptoms were ascertained as a binary variable using the self-completed Edinburgh Postnatal Depression Scale (EPDS), the most widely used screening instrument for perinatal depression in international and Turkish research. It focuses primarily on cognitive rather than somatic symptoms of depression although does include a question on sleep.^15^ It is a 10-item self-report measure with a 4-point Likert scale for each item, scored between 0 and 3, giving a potential scale score range of 0–30. Its reliability and validity in Turkish have been established in two independent studies, both recommending a cut-off of ≥13 to define caseness, and the same was applied in our study to define case-level depressive symptoms (hereafter referred to as ‘depression’ for brevity).^16^ The reliability and validity study of the scale in Turkish was established by Aydın *et al*^16^ using the SCID as a gold standard, finding sensitivity and specificity of 0.76 and 0.71, respectively, and a Cronbach's α value of 0.72. In another validation study in Turkey by Engindeniz (1996), sensitivity was found to be 0.84 and specificity 0.88.^16^

Quality of family relationships was measured using the Close Persons Questionnaire (CPQ).^17^ This is a widely applied instrument which focuses on three aspects of the quality of individual relationships—(1) emotional support; (2) practical support; (3) negative aspects of the relationship. In a departure from the standard application of this instrument (where participants are asked to choose their most salient relationships to be rated), the index relationships were imposed so that questions were asked specifically and solely about the spouse, mother and mother-in-law.

Other covariates in this analysis were as follows: (1) age (18–22, 23–25, 26–29 and 30+ years), (2) number of living children (0, 1 and 2+), (3) education level (5 years or less, 6–8, 9–11 and 12+ years), (4) family income (630 Turkish Liras (TRY) or less, 631–900 TRY, 901–1400 TRY and 1401+ TRY), (5) general physical health (very good, good and average/bad/very bad), (6) life stressors/events over the 12 months prior to the baseline assessment (from a checklist enquiring about debt, hunger from lack of food, recent separation, problems with friends, recent illness/assault, violence to self, illness in a relative, death of a close family member, death of another relative, problems with a job, problems with money, problems with the justice system, any robbery and categorising these event according to the number of life events: 0, 1, 2 and 3 or more), (7) history of any emotional problems (two categories: yes or no; based on self-reported information to an open-ended non-specific question, seeking to elicit common mental health problems regardless of formal diagnosis), (8) child health (three categories: very good, good and average/bad/very bad) and (9) family structure (two categories: nuclear or traditional).

Family structure was considered as both an independent variable and an effect modifier and was defined as a binary variable, categorising nuclear and traditional/extended family structure. A nuclear family structure was defined as a wife and husband living alone or with their children in the same household, whereas a traditional/extended family structure was defined if at least one other adult was living with the married couple in the same household. In Turkish society this would nearly always be the husband's mother or father.

Physical abuse had been asked about (‘Have you been beaten, injured or violated by someone during the pregnancy or the past year?’); however, reported prevalences at the baseline and follow-up examinations were only 2.5% and 2.4% respectively, precluding its further analysis. In addition, very few (1.8%) reported smoking and none reported alcoholism or substance abuse since pregnancy. These variables were therefore not considered further as covariates.

### Statistical analysis

A target sample size of 750 women was calculated with the prospective study in mind. This assumed a prevalence of 25% for case-level depression on the chosen scale at baseline, a persistence rate of 30% through to the postnatal period, and aimed to detect a 0.5 SD group difference in mean score for a given quality of relationship measure between persistent and non-persistent groups at 80% power (α 0.05, two-sided test). At the same level of power, this sample size was calculated as allowing the detection of a 0.3 SD group difference between participants with and without case-level depression at baseline, assuming a more conservative 13% prevalence.

Postnatal prevalence, incidence and persistence of depression were calculated followed by unadjusted analyses of associations with covariates. Incidence of depression was calculated in women who were not depressed at baseline based on proportions of new cases at follow-up in that sample. Persistence of depression was calculated in women who were depressed at baseline based on the proportions with depression at follow-up. For primary analyses, caseness on the EPDS was analysed as a binary dependent variable with CPQ subscales as independent variables. In secondary analyses, in order to take advantage of the continuously distributed data on social support, the CPQ subscales were treated as dependent variables (ie, testing the differences in mean scores for baseline social support between cases of incident/persistent depression and controls at follow-up). t Tests were initially used to investigate significance and then linear regression models were used to adjust for covariates. The sample size was felt to be sufficient to justify this approach of linear modelling, despite non-normal CPQ subscale distributions. Stratified analyses were used to investigate effect modification by family structure with interaction terms re-tested in linear regression models.

## Results

### Sample characteristics

Of 730 participants assessed in their third trimester (95% of those approached about the study), 578 (79%) were reassessed between 2–6 months after childbirth. The main reason for loss to follow-up (16%) was migration of families due to re-allocation of housing in certain areas and consequent loss of contact. Thirty-seven (5%) previous participants refused follow-up assessment. In terms of baseline characteristics between two groups who were assessed and not assessed at follow-up examination, there were no significant differences in terms of age, education level, family setting, depression or any social support measure. Specifically, baseline depression prevalence was 32.6% in those followed and 31.5% in those not followed (χ^2^ 0.14, df 1, p=0.61). However, proportions with no, one, or two or more previous children were 48.9%, 34.5% and 16.6% in women followed, compared to 63.6%, 24.5% and 12% respectively in women not followed (χ^2^ 17.04, df 2, p=0.002).

Participants’ mean age at baseline was 26.1 years (SD 5.2, range 18–44), and their mean education duration was 8.4 years (SD 3.7). Regarding social structure, 67% lived in a nuclear family. Participants in extended family settings lived with either their mother-in-law (27%) or father-in-law (21%) or both. Almost all (97%) participants were married and all of these, apart from two, lived with their husband. The majority (88%) of participants reported a ‘good’ or ‘very good’ relationship with their husband, and 60% reported that the husband helped take care of the baby. In terms of the index pregnancy and childbirth, 79% reported that the pregnancy was planned and 49% had no living children at the time of enrolment. Almost all of the participants (99%) gave birth at health facilities and 64% had a natural delivery. All participants gave birth to a live baby. There were equal numbers of live male (50.5%) and female babies. Two participants gave birth to twins and one baby died after birth; 97% of babies were vaccinated before the follow-up interview.

### Incidence and persistence of depression from antenatal to postnatal examinations

Of 730 participants assessed at baseline, 238 (32.6%) had total EPDS scores ≥13. Of the 578 participants followed up at 2–6 months postpartum, 151 (26.1%) had EPDS scores above this cut-off. Of those followed successfully (n=578), 55 of the 386 without antenatal depression had depression at follow-up, indicating case incidence of 13.9%. Ninety-six of the 192 cases with antenatal depression within the total sample (n=578) continued to have depression at postnatal assessment indicating case persistence of 49.7%. Of the 151 postnatal cases, 90 (59.6%) had previously screened positively for antenatal depression.

### Social support associations with incidence of depression

Unadjusted analyses are summarised for case incidence in table 1. Of the covariates investigated, only lower family income and adverse life events were significantly associated with case incidence. No association was found between time since the index childbirth and case incidence (OR per month increment 1.05; 95% CI 0.95 to 1.17; table 1). Associations of social support at baseline with case incidence are summarised in table 2. In unadjusted models, depression incidence was significantly associated with lower support at the baseline from the husband and mother-in-law on emotional subscales. After full adjustment, lower emotional support from the mother-in-law remained associated with incidence and the association with spouse support was substantially confounded.

**Table 1:** Unadjusted associations between participant characteristics and incidence of case level depressive symptoms (ie, restricted to non-cases at baseline) N=354

	N	Depression incidence % (n)	ORs (CIs)	χ^2^ (df), p Value
Age				0.14 (1), p=0.71
18–22	91	15.4 (14)	Reference	
23–25	77	13.0 (10)	0.82 (0.34 to 1.97)	
26–29	102	9.8 (10)	0.60 (0.25 to 1.42)	
30–44	84	19.0 (16)	1.29 (0.59 to 2.85)	
Number of children				1.56 (1), p=0.21
0	182	13.2 (24)	Reference	
1	129	10.9 (14)	0.80 (0.40 to 1.62)	
≥2	52	23.1 (12)	1.98 (0.91 to 4.29)	
Reported health of the baby				0.72 (1), p=0.40
Very good	159	16.4 (26)	Reference	
Good	189	11.1 (21)	0.64 (0.35 to 1.19)	
Average, bad, very bad	15	20.0 (3)	1.28 (0.34 to 4.85)	
Education duration (year)				2.40 (1), p=0.12
≤5	121	17.4 (21)	Reference	
6–8	74	14.9 (11)	0.83 (0.38 to 1.84)	
9–11	118	11.9 (14)	0.64 (0.31 to 1.33)	
12≥	43	9.3 (4)	0.49 (0.16 to 1.51)	
Family income (TRY)				7.21 (1), p=0.01*
≤630	83	21.7 (18)	Reference	
631–900	91	15.4 (14)	0.66 (0.30 to 1.42)	
901–1400	121	9.9 (12)	0.40 (0.18 to 0.88)	
1401–23 000	51	7.8 (4)	0.31 (0.09 to 0.97)	
Physical health				2.55 (1), p=0.11
Very good	62	8.1 (5)	Reference	
Good	245	14.3 (35)	1.90 (0.71 to 5.07)	
Average/bad/very bad	55	18.2 (10)	2.53 (0.81 to 7.94)	
Number of life events/stressors				5.71 (1), p=0.022*
0	180	10.0 (18)	Reference	
1	91	20.9 (19)	2.38 (1.17 to 4.79)	
2	39	20.5 (8)	2.32 (0.93 to 5.81)	
3+	18	22.2 (4)	2.57 (0.76 to 8.65)	
				
Emotional problems in the past				2.05 (1), p=0.15
No	226	12.4 (28)	Reference	
Yes	122	18.0 (22)	1.56 (0.85 to 2.85)	
Family structure				1.91 (1), p=0.18
Nuclear	245	15.5 (38)	Reference	
Traditional	118	10.3 (12)	0.62 (0.31 to 1.23)	

**Table 2:** Logistic regression models of associations between baseline social support measures (independent variables) and depression incidence (dependent variable; n=354)

	Associations between baseline social support and depression incidence (ORs per unit increase in baseline social support measure, 95% CI)		
	Emotional	Practical	Negative aspects
	From husband		
Unadjusted	0.90 (0.85 to 0.96)*	0.92 (0.80 to 1.05)	1.13 (0.98 to 1.32)
Adjusted	0.95 (0.87 to 1.03)	1.06 (0.88 to 1.26)	1.02 (0.87 to 1.19)
	From mother		
Unadjusted	0.96 (0.91 to 1.01)	1.02 (0.91 to 1.13)	1.03 (0.88 to 1.19)
Adjusted	0.97 (0.91 to 1.04)	1.08 (0.94 to 1.23)	1.04 (0.88 to 1.23)
	From mother in-law		
Unadjusted	0.91 (0.86 to 0.96)*	0.92 (0.82 to 1.02)	0.92 (0.79 to 1.06)
Adjusted	0.93 (0.87 to 0.99)*	0.95 (0.83 to 1.09)	0.95 (0.80 to 1.13)

Adjusted for age, number of children, duration of education, family income, reported health of the baby, reported physical health, previous emotional problems, number of life stressors/events and timing of follow-up. *p<0.05.

### Social support associations with persistence of depression

Unadjusted analyses are summarised for case persistence in table 3. Of the covariates investigated, only the number of adverse life events was associated with case persistence (table 3). No association was found between time since the index childbirth and case persistence (OR per month increment 1.03; 95% CI 0.92 to 1.15). Associations of social support at baseline with case persistence are summarised in table 4. Depression persistence was significantly associated with lower support from the husband on the emotional subscale which remained significant after full adjustment.

**Table 3:** Unadjusted associations between participant characteristics and persistence of case level depressive symptoms (ie, restricted to cases at baseline) N=181

	n	Depression prevalence% (n)	ORs (CIs)	χ^2^ (df), p Value
Age				0.01 (1), p=0.97
18–22	49	51.0 (25)	Reference	
23–25	51	54.9 (28)	1.17 (0.53 to 2.57)	
26–29	32	31.3 (10)	0.44 (0.17 to 1.11)	
30–44	49	57.1 (28)	1.28 (0.58 to 2.84)	
Number of children				1.42 (1), p=0.71
0	85	50.6 (43)	Reference	
1	58	48.3 (28)	0.91 (0.47 to 1.79)	
≥2	39	51.3 (20)	1.03 (0.48 to 2.20)	
Reported health of the baby				2.07 (1), p=0.15
Very good	73	45.2 (33)	Reference	
Good	97	52.6 (51)	1.34 (0.73 to 2.47)	
Average, bad, very good	11	63.6 (7)	2.12 (0.57 to 7.88)	
Education duration (year)				2.70 (1), p=0.10
≤5	61	41.0 (25)	Reference	
6–8	31	54.8 (17)	1.75 (0.73 to 4.18)	
9–11	65	50.8 (33)	1.49 (0.73 to 3.01)	
12≥	17	64.7 (11)	2.64 (0.86 to 8.08)	
Family income (TRY)				1.60 (1), p=0.21
≤630	48	43.8 (21)	Reference	
631–900	52	50.0 (26)	1.29 (0.59 to 2.83)	
901–1400	46	45.7 (21)	1.08 (0.48 to 2.44)	
1401–23 000	23	65.2 (15)	2.41 (0.86 to 6.75)	
Physical health				0.11 (1), p=0.74
Very good	30	43.3 (13)	Reference	
Good	99	55.6 (55)	1.64 (0.72 to 3.72)	
Average/bad/very bad	53	43.4 (18)	1.00 (0.41 to 2.47)	
Number of life events/stressors				4.78 (1), p=0.03*
0	56	41.1 (23)	Reference	
1	44	52.3 (39)	1.57 (0.71 to 3.48)	
2	26	42.3 (11)	1.05 (0.41 to 2.70)	
3+	33	60.6 (20)	2.21 (0.92 to 5.31)	
Emotional problems in the past				2.12 (1), p=0.14
No	56	39.3 (22)	Reference	
Yes	123	55.3 (68)	1.91 (1.00 to 3.64)	
Family structure				0.02 (1), p=0.88
Nuclear	119	49.6 (59)	Reference	
Traditional	63	50.8 (32)	1.05 (0.57 to 1.94)	

**Table 4:** Logistic regression models presenting adjusted associations between baseline social support measures (independent variables) and depression persistence (dependent variable; n=181)

	Associations between baseline social support and depression persistence (ORs per unit increase in baseline social support measure, 95% CI)		
	Emotional	Practical	Negative aspects
	From husband		
Unadjusted	0.91 (0.86 to 0.96)*	0.94 (0.83 to 1.06)	1.12 (0.97 to 1.29)
Adjusted	0.89 (0.83 to 0.96)*	0.96 (0.83 to 1.13)	1.12 (0.93 to 1.35)
	From mother		
Unadjusted	0.97 (0.92 to 1.01)	0.99 (0.89 to 1.09)	1.03 (0.88 to 1.19)
Adjusted	0.95 (0.89 to 1.02)	0.99 (0.87 to 1.15)	1.16 (0.95 to 1.42)
	From mother in-law		
Unadjusted	0.97 (0.92 to 1.02)	0.97 (0.88 to 1.07)	1.09 (0.98 to 1.22)
Adjusted	0.98 (0.91 to 1.05)	1.01 (0.89 to 1.16)	1.13 (0.98 to 1.31)

Adjusted for age, number of children, duration of education, family income, reported health of the baby, reported physical health, previous emotional problems, number of life stressors/events and timing of follow-up. *p<0.05.

### Social support associations between family structure and depression incidence or persistence

Regarding family structure type, there was no direct association with either depression incidence or persistence (tables 1–4) and there were no significant interactions between depression status (either incidence or persistence) and family structure in the linear regression models of social support subscales (table 5–8).

**Table 5:** Effect modification by family structure for the association between incidence/persistence of depression and baseline social support (n=547)

Depression outcome	Interaction terms between social support and traditional family structure (ORs, 95% CIs, p values)		
	Emotional	Practical	Negative aspects
	From husband		
Incidence	0.99 (0.97 to 1.02), p=0.58	0.96 (0.89 to 1.03), p=0.23	0.95 (0.89 to 1.01), p=0.11
Persistence	1.00 (0.98 to 1.03), p=0.44	1.01 (0.95 to 1.09), p=0.71	1.02 (0.97 to 1.08), p=0.43
	From mother		
Incidence	0.99 (0.96 to 1.02), p=0.40	0.96 (0.89 to 1.03), p=0.24	0.95 (0.84 to 1.06), p=0.35
Persistence	0.99 (0.97 to 1.02), p=0.77	1.01 (0.94 to 1.08), p=0.80	1.02 (0.92 to 1.13), p=0.71
	From mother in-law		
Incidence	1.01 (0.97 to 1.05), p=0.77	0.97 (0.88 to 1.06), p=0.44	0.96 (0.89 to 1.03), p=0.24
Persistence	1.02 (0.98 to 1.06), p=0.40	1.03 (0.94 to 1.13), p=0.50	0.99 (0.94 to 1.05), p=0.81

**Table 6:** Linear regression models presenting adjusted associations between depression incidence (independent variable) and baseline social support measures (dependent variables; n=354)

Nature of support	Association with depression incidence (B-value, 95% CI)			
	Unadjusted	Model 1	Model 2	Model 3
From husband				
Emotional	−2.4 (−3.8 to −1.1)*	−2.4 (−3.7 to −1.1)*	−1.5 (−2.8 to −0.2)*	−1.1 (−2.4 to 0.2)
Practical	−0.4 (−1.0 to 0.3)	−0.4 (−1.0 to 0.3)	−0.1 (−0.8 to 0.6)	0.1 (−0.6 to 0.7)
Negative aspects	0.5 (−0.1 to 1.1)	0.5 (−0.1 to 1.1)	0.5 (−0.2 to 1.1)	0.3 (−0.3 to 1.0)
From mother				
Emotional	−1.3 (−3.0 to 0.4)	−1.3 (−2.9 to 0.4)	−1.0 (−2.7 to 0.7)	−0.8 (−2.4 to 0.9)
Practical	0.1 (−0.8 to 1.0)	−0.2 (−0.7 to 1.0)	0.2 (−0.7 to 1.1)	0.5 (−0.5 to 1.4)
Negative aspects	0.1 (−0.5 to 0.7)	0.1 (−0.5 to 0.7)	0.2 (−0.5 to 0.8)	0.2 (−0.5 to 0.9)
From mother in law				
Emotional	−3.4 (−5.4 to −1.3)*	−3.4 (−5.3 to −1.4)*	−2.6 (−4.6 to −0.5)*	−2.8 (−4.9 to −0.8)*
Practical	−0.8 (−1.7 to 0.2)	−0.8 (−1.7 to 0.2)	−0.5 (−1.4 to 0.5)	−0.4 (−1.4 to 0.6)
Negative aspects	−0.4 (−1.1 to 0.3)	−0.4 (−1.1 to 0.3)	−0.2 (−1.0 to 0.5)	−0.3 (−1.0 to 0.5)

Model 1 Adjusted for age.

Model 2 Adjusted for 1 and number of children, duration of education, family income, baby health, physical health and previous emotional problems.

Model 3 Adjusted for 2 and number of life stressors/events and timing of follow-up.

*p<0.05.

**Table 7:** Linear regression models presenting adjusted associations between depression persistence (independent variable) and baseline social support measures (dependent variables)

Nature of support	Association with depression persistence (B-value, 95% CI)			
	Unadjusted	Model 1	Model 2	Model 3
From husband				
Emotional	−3.5 (−5.2 to −1.6)*	−3.4 (−5.2 to −1.7)*	−3.4 (−5.4 to −1.4)*	−3.2 (−5.2 to −1.3)*
Practical	−0.4 (−1.1 to 0.4)	−0.2 (−1.1 to 0.4)	−0.2 (−1.0 to 0.7)	−0.3 (−1.1 to 0.6)
Negative aspects	0.5 (−0.2 to 1.1)	0.5 (−0.1 to 1.1)	0.5 (−0.2 to 1.1)	0.5 (−0.3 to 1.2)
From mother				
Emotional	−1.4 (−3.3 to 0.6)	−1.5 (−3.4 to 0.5)	−1.6 (−3.7 to 0.6)	−1.3 (−3.5 to 1.0)
Practical	−0.1 (−1.0 to 0.8)	−0.1 (−1.0 to 0.8)	−0.7 (−1.0 to 0.9)	−0.1 (−1.1 to 0.9)
Negative aspects	0.1 (−0.5 to 0.8)	0.1 (−0.5 to 0.8)	0.2 (−0.5 to 1.0)	0.4 (−0.4 to 1.2)
From mother in law				
Emotional	−1.2 (−3.1 to 0.7)	−1.2 (−3.1 to 0.7)	−1.0 (−2.9 to 1.0)	−0.4 (−2.5 to 1.7)
Practical	−0.3 (−1.2 to 0.7)	−0.3 (−1.2 to 0.6)	−0.3 (−1.3 to 0.7)	0.2 (−0.9 to 1.2)
Negative aspects	0.8 (−0.1 to 1.7)	0.8 (−0.2 to 1.7)	0.7 (−0.3 to 1.6)	0.7 (−0.3 to 1.7)

Model 1 Adjusted for age.

Model 2 Adjusted for 1 and number of children, duration of education, family income, baby health, physical health and previous emotional problems.

Model 3 Adjusted for 2 and number of life stressors/events and timing of follow-up.

*p<0.05.

**Table 8:** Effect modification by family structure for the association between incidence/persistence of depression and baseline social support

Dependent variable (social support)	Interaction terms between depression and traditional family structure (B coefficients, 95% CIs, p values) adjusted for all covariates (Model 3 from table 6)	
	Depression incidence	Depression persistence
From husband		
Emotional	1.15 (−1.79 to 4.10), p=0.44	−1.11 (−4.95 to 2.74), p=0.57
Practical	0.23 (−1.28 to 1.73), p=0.77	0.18 (−1.57 to 1.92). p=0.84
Negative aspects	−1.50 (−3.04 to 0.04), p=0.06	1.03 (−0.47 to 2.54). p=0.18
From mother		
Emotional	0.52 (−3.25 to 4.29), p=0.79	−1.12 (−5.64 to 3.40), p=0.62
Practical	0.73 (−1.34 to 2.81), p=0.49	−1.47 (−3.52 to 0.58). p=0.16
Negative aspects	−0.20 (−1.75 to 1.36), p=0.80	0.74 (−0.88 to 2.36), p=0.37
From mother in law		
Emotional	0.39 (−1.73 to 2.52), p=0.72	1.04 (−3.38 to 5.46), p=0.64
Practical	0.47 (−1.89 to 2.83), p=0.69	0.30 (−1.81 to 2.40), p=0.78
Negative aspects	0.18 (−1.55 to 1.90), p=0.84	−1.07 (−3.18 to 1.05), p=0.32

### Sensitivity analyses

Additional analyses were carried out, as mentioned, modelling baseline social support measures as continuous dependent variables against incident/persistent depression as binary independent variables in linear regression models (tables 6–8). In summary, the findings were generally comparable—women with incident depression had previously reported lower emotional support from the mother-in-law, those with persistent depression had previously reported lower emotional support from the husband, and there were no significant interaction terms with family structure.

## Discussion

In a large cohort of Turkish women living in mixed nuclear/traditional family settings followed from examinations carried out during their third trimester to between 2 and 6 months following childbirth, incident postnatal depression occurred in 13.9% of women without antenatal depression. Depression persisted from the antenatal to postnatal period in 49.7%. Some measures of family support were associated with these outcomes (lower emotional support from the husband with persistence of depression; lower emotional support from the mother-in-law with incidence of depression), although most were not. No direct or modifying role of family structure was found for either outcome.

### Incidence and persistence of perinatal depression

It is increasingly recognised that many cases of perinatal depression begin in the antenatal period and persist after childbirth.^2^ Antenatal and postnatal depression have also been reported to share similar prevalences to those for depression in the general population with estimates ranging from 12% to 20%, with a commonly reported estimate of 13%.^2^ The prevalences of antenatal and postnatal common mental disorder in low-income and middle-income countries have been estimated at 15.6% and 19.8%, respectively.^7^ The reported prevalence of postnatal depression in Turkey has been substantially higher (34.6%) than that in many western countries,^16^ and similarly high antenatal prevalences have also been found (33.1% and 27.5%).^11^ ^18^ As has been discussed in a previous report on baseline findings in this cohort,^11^ the prevalence of antenatal depression was comparable to findings in other Turkish antenatal samples using this scale and cut-off,^18^ although higher than findings from other countries: in particular, higher than the 12.8% prevalence in the third trimester concluded in a meta-analysis of studies undertaken in developed countries^19^ and the 18.4% period prevalence for pregnancy in another meta-analysis.^20^ The prevalence of postnatal depression was also comparable to findings in other Turkish postnatal samples using this scale and cut-off,^16^ although also higher than findings from other countries and compared to the 12% prevalence reported in a systematic review of studies undertaken in developed countries in all but one instance.^19^ ^21^ ^22^ Although this could reflect differences in scale performance and appropriate cut-offs, underlying inequalities in national or regional risk do need further evaluation.

Evidence on the evolution of depression from antenatal to postnatal periods is more limited.^20^ The incidence of depression in the postnatal period has been previously reported as ranging from 6.8 to –20.1%, albeit over different follow-up periods and time points during the postnatal period, as well as different countries.^20^ The incidence of postnatal depression in our sample (13.9%) was similar to that reported by Areias *et al*^23^ in Portuguese women, of whom 14.5% had a new episode during the first 3 months of the postnatal period. The persistence of depression between antenatal and postnatal examinations (49.7%) was similar to the 49.6% found in an Iranian cohort, but slightly higher than the 43.7% reported in a British study.^4^ ^24^

In the recent review by Fisher *et al*^7^, strong predictors of perinatal common perinatal mental disorders included psychiatric history, being socially and economically disadvantaged, gender-based factors, role restrictions regarding housework and infant care, as well as lower perceived social support. Stressful life events were associated with both incidence and persistence of depression in the cohort described here. However, this exposure was not the focus for the analysis and its independence was not assessed. In unadjusted analyses, lower income predicted incidence but not persistence of depression. Otherwise, no other covariates were associated with incidence or persistence, although having two or more previous children; worse reported physical health and past emotional problems had been found to be significantly associated with depression at baseline.^11^

### Family relationships and perinatal depression

The quality of family relationships is important in the aetiology of depression in women, particular depression occurring around childbirth. As reviewed by Fisher *et al*,^7^ the quality of a woman's intimate partner relationship has been found to be closely related to her perinatal mental health in low-income and low-middle income countries as in high-income countries. Family relationships may well be particularly salient in societies where there are relatively high expectations of family support at times of crisis. Studies focusing on conflicts between a woman and her in-laws have identified a raised risk of perinatal mental disorder associated with insufficient social support.^7^ These support structures are coming increasingly under strain with rapid demographic transitions in many countries; however, the impact of these transitions on women's mental health has received relatively little investigation. This should be seen in the context of wider gender inequalities, especially concerning economic opportunities,^25^ which may adversely affect women's mental health. As mentioned, Turkish society has distinct advantages for research of this nature because of the longstanding coexistence of traditional Middle Eastern family structures with the nuclear structures more commonly seen in Western settings. Furthermore, women in Turkey are largely free to choose their living arrangements with minimal stigma associated with either model. However, Turkish society still supports traditional household roles for women such as preparation of meals and taking care of children.^25^ The relationships between women, their mother and their mother in-law remain important in Turkish culture, whether the woman is living in a nuclear or extended family setting.^26^ A woman in a traditional setting will typically move to live with her husband and his family in the same house when she gets married. In this setting, the expected role of a woman's own mother is to support this marriage by helping her daughter on practical issues (eg, taking care of children) and emotional issues. To our knowledge, ours is the first prospective study of perinatal depression and family support in a Middle Eastern society, although this has been previously investigated in a cross-sectional study of postnatal depression in Turkey.^9^

In our prospective analyses, the incidence and persistence of depression were associated with lower emotional support from the mother-in-law and the husband, respectively. On the other hand, no predictive associations were found for practical support or negative aspects of the relationships. While this might reflect different psychometric properties between the subscales, it might also reflect more salient features of the relationship quality for participants. Overall relationships with the husband were reported as good, which might have reduced our ability to detect an association. As reviewed by Fisher *et al*,^7^ several studies have investigated the risks associated with difficult interpersonal relationships, including with in-laws, in low-income middle-income countries. In Bangladesh, poor relationship with and low support from the mother-in-law were associated with postnatal depression,^27^ and incidence of postnatal depression was associated with problems with in-laws in India,^28^ although social support did not predict persistence of postnatal depression in Pakistan;^29^ however, none of these studies evaluated social support with a structured instrument. Fisher *et al*^30^ used the 24-item Intimate Bond measure to assess the quality of the intimate relationship and its association with antenatal and postnatal perinatal depression in Vietnam, but the cross-sectional nature of study limits conclusions regarding direction of causation.

The associations with support from the mother-in-law rather than the mother (both in cross-sectional associations with antenatal depression previously reported,^11^ and with prospective data here) may reflect the relative importance of the former relationship in Turkish society. Another explanation might be that the perceived relationship with the mother-in-law is more strongly linked with the quality of a woman's marriage. Finally, it could reflect reluctance on the part of participants to report problems with parental relationships, particularly emotional relationships. Support from family members has been found to be an important buffer against depression in women from other low and middle-income settings,^31^ and some research in Islamic nation settings has suggested both high prevalence of perinatal disorder and a potentially harmful role of disruptions to traditional family structures.^21^

### Family structure and perinatal depression

No difference was found at baseline in prevalence of antenatal depression between traditional and nuclear family settings^11^ and our findings of similar incidence and persistence rates are consistent with this. At baseline, lack of emotional support from the husband was found to be more strongly associated with antenatal depression in traditional compared to nuclear family settings but no significant interactions with family structure were found for depression incidence or persistence in these prospective analyses. This appears to suggest that the importance of the family environment remains consistent across different family structures, to the extent that these could be characterised and quantified in this setting, and within the statistical power derived from the sample size. Our findings are consistent with the lack of association found between nuclear family settings and postnatal depression in Bangladesh.^27^ Extended families were protective in a Pakistan study of antenatal and postnatal depression, particularly support from family members with routine child-care and the presence of the infant's grandmother, although analyses were cross-sectional.^21^

### Clinical implications

As mentioned, we believe that this study is the most extensive prospective investigation of the relationship between family support and perinatal depression in a Middle Eastern context. In general, prospective associations with depression incidence/persistence, although identified, were not as strong or consistent as those previously observed in this sample with prevalent antenatal depression, and modification by family structure was not as marked either. One potential reason for this is that the cross-sectional association between lower social support and depression is primarily accounted for by negative effects of depression on social support, rather than effects of low social support on depression risk; however, this requires further evaluation. The relatively high incidence and persistence rates do indicate that clinical services should focus on the full perinatal period, rather than predominantly on the postnatal period, and that preventative interventions should be considered and evaluated for women with emerging depressive symptoms in pregnancy.

### Strengths and limitations

Strengths of this study include its prospective design and the particular features of the setting, as previously mentioned, generating a large and heterogeneous sample. Follow-up rates were reasonable and refusal rates low, reducing the risk of selection bias. In addition, baseline characteristics did not differ substantially between those present or not at follow-up—most importantly, attrition was not predicted by depression or social support at baseline, although there were some differences in numbers of previous children between those followed and not followed, as previously described.

Statistical power should also be considered, although post hoc power calculations indicated 80% power to detect modest effect sizes in the analysed samples (with respect to differences in mean social support scale scores) of 0.6 SD for depression incidence analyses and 0.4 SD for those of depression persistence (α 0.05). However, smaller effects cannot be excluded. Furthermore, there was a relatively broad range of follow-up intervals which might have obscured factors operating over defined time periods. Although the distribution of follow-up intervals was broad, the timing of follow-up was not associated with either incidence or persistence of depression. A comprehensive range of covariates were taken into account, reducing the likelihood of confounding, although this cannot be ruled out entirely. For example, no account was taken of the woman's own upbringing and family structure.

Considering the outcome, the EPDS has been widely used in international research; however, it should be borne in mind that it is a screening instrument, measuring number of depressive symptoms and not seeking to define specific depression syndromes or to apply diagnostic criteria. While different associations with incidence and persistence of depression might reflect type 1 error, given the number of analyses, the individual associations (with emotional support from the spouse/mother-in-law) are consistent with baseline cross-sectional associations. In panel surveys of older community populations, several studies have found different predictors of incidence and persistence of depression—for example physical ill health primarily predicting incidence and social support primarily predicting persistence.^32^ Our findings suggest that the relationship with the mother-in-law may preferentially affect a woman's risk of developing depression in the postnatal period, possibly reflecting alterations in family dynamics following childbirth. For women who are depressed in the antenatal period, on the other hand, the quality of the relationship with their husband appears to be the most salient factor in predicting recovery or not after childbirth.

Considering family structure, while it is our belief that the different structures characterised represent considerable heterogeneity in experience, it is possible that there are societal norms and expectations in Turkey which transcend these structural differences (eg, pertaining to the importance of the mother-in-law relationship even where there is no coresidence). Also, it is important to bear in mind that the nuclear and traditional family structures investigated in this study have coexisted over a long period in Turkey. Findings therefore may not generalise to other societies where nuclear families are a relatively recent phenomenon and potentially less supported or more stigmatised.
